# Bright light treatment of depression for older adults [ISRCTN55452501]

**DOI:** 10.1186/1471-244X-5-41

**Published:** 2005-11-09

**Authors:** Richard T Loving, Daniel F Kripke, Jeffrey A Elliott, Nancy C Knickerbocker, Michael A Grandner

**Affiliations:** 1 Department of Psychiatry, University of California, San Diego, USA

## Abstract

**Background:**

The incidence of insomnia and depression in the elder population is significant. It is hoped that use of light treatment for this group could provide safe, economic, and effective rapid recovery.

**Methods:**

In this home-based trial we treated depressed elderly subjects with bright white (8,500 Lux) and dim red (<10 Lux) light for one hour a day at three different times (morning, mid-wake and evening). A placebo response washout was used for the first week. Wake treatment was conducted prior to the initiation of treatment, to explore antidepressant response and the interaction with light treatment. Urine and saliva samples were collected during a 24-hour period both before and after treatment and assayed for aMT6s and melatonin respectively to observe any change in circadian timing. Subjects wore a wrist monitor to record light exposure and wrist activity. Daily log sheets and weekly mood (GDS) and physical symptom (SAFTEE) scales were administered. Each subject was given a SCID interview and each completed a mood questionnaire (SIGH-SAD-SR) before and after treatment. Also, Hamilton Depression Rating (SIGH-SAD version) interviews were conducted by a researcher who was blind to the treatment condition. A control group of healthy, age-matched, volunteers was studied for one day to obtain baseline data for comparison of actigraphy and hormone levels.

**Results:**

Eighty-one volunteers, between 60 and 79 years old, completed the study. Both treatment and placebo groups experienced mood improvement. Average GDS scores improved 5 points, the Hamilton Depression Rating Scale (HDRS) 17 scores (extracted from the self-rated SIGH-SAD-SR) improved 6 points. There were no significant treatment effects or time-by-treatment interactions. No significant adverse reactions were observed in either treatment group. The assays of urine and saliva showed no significant differences between the treatment and placebo groups. The healthy control group was active earlier and slept earlier but received less light than the depressed group at baseline.

**Conclusion:**

Antidepressant response to bright light treatment in this age group was not statistically superior to placebo. Both treatment and placebo groups experienced a clinically significant overall improvement of 16%.

## Background

Several reviews have emphasized the enormous socioeconomic impact in elders of insomnia and depression which are often intertwined [1-5]. Reasons for depression in elders include: loss of body strength, health and autonomy, loss of loved ones and friends, loss of occupational status, and fear of impending death [6-8]. Some years ago, the suicide rate was thought to be low among the oldest adults, but this rate has been rising [9,10], despite the improved financial condition of the over-60 population. Antidepressant drugs are reasonably effective in older patients, but depression often is inadequately-treated or chronic. In elders, there is a greater risk of medication side effects such as falls, over-sedation, and anti-cholinergic disturbances [11,12]. These difficulties point to the potential usefulness of light treatment for depressed elders either to supplement or replace pharmacotherapy. Development of new light treatments could have widespread benefit for millions of aging Americans with insomnia and depression.

Light treatment for seasonal depression (SAD) has become accepted in the Clinical Practice Guidelines issued by the U.S. Department of Health and Human Services [13] and the American Psychiatric Association's *Treatment of Psychiatric Disorders *[14]. It is gratifying to see this safe, inexpensive, rapid and effective treatment spreading worldwide into clinical practice. Fewer light treatment studies have been conducted to investigate nonseasonal major depressions, but light is effective for nonseasonal depression [15]. In nonseasonal depression, light treatment may produce as much incremental benefit or more when antidepressant drugs are also administered [15]. There has been little formal study of light treatment in the elderly, partly because seasonal depression seems less common among women after menopause [16].

Several small studies have suggested promising results with bright-light treatment of elderly patients who have distinctive phase-advanced sleep disturbances [17], or with dementia [18-22], but there has been little critical examination of the value of bright light for the majority of elderly Americans with less specific mood or sleep complaints.

The mechanisms by which light produces antidepressant effects are of essential scientific interest, both for understanding the underlying etiologies of depressive illnesses and for guiding our treatment strategies. There is considerable evidence that morning light is better than evening light for SAD [15,23-26], but some studies have found little difference between timings [27,28], and the apparent advantage of morning light may be partly explained by an anomalous order effect in cross-over designs [29]. When morning light is effective, it might work by suppressing or phase-advancing an overly-late melatonin offset [30-32]. It seems possible (though unproven) that conditions in which sleep complaints are most prominent and conditions in which mood complaints are most prominent might have a common etiology in circadian phase malsynchronization which is characterized by abnormal entrainment of circadian rhythms to the solar day and/or abnormal relationships among rhythms in the body.

Phase-typing as a predictor of light-treatment response was proposed by Lewy and colleagues [33] and has been previously employed to select light treatment timing prospectively [34]. We used this method in the belief that without phase-typing and individualized treatment assignment, sub-optimal results would be obtained.

Our early studies [35,36] the Praskos' study [37], and especially the work of Neumeister et al. [38], Loving [39], and Bloching [40] suggested that partial sleep deprivation combined with bright light produces remarkable antidepressant responses, as demonstrated by dramatic contrasts between bright light and placebo. Considering the evidence that sleep deprivation may tend to accentuate the contrast of bright light and placebo, we expected partial sleep deprivation would add to the potential effectiveness of individual timed light therapy.

In this paper, we report on a clinical trial of bright light treatments in the home, with individualized treatment timing. The goals of the study were to determine if light resistance in the 60–79 year age range can be overcome with 4 weeks of bright light treatment and to gain information on the relative benefit of different times of light treatment.

Our aims were to compare the effectiveness of morning, mid-day, and evening light treatments, individualized according to clinical and actigraphic assessments, in correcting circadian disturbances. Also, the trial sought to demonstrate greater improvements in mood and sleep among those volunteers receiving 4 weeks bright light as contrasted to placebo.

## Methods

Recruitment from July 2000 to December 2003 used advertising and community presentations. Written informed consent was obtained from each participant prior to the start of the study, in accordance with the guidelines set forth by the Declaration of Helsinki. The study protocol and consent form were approved by the UCSD Human Research Protection Program. In addition, those participants who were being treated for depression by either a physician or counselor were requested to obtain the written agreement of the therapist for the study, to assure that there was no interference with ongoing treatment and treatment responsibility. Patients were encouraged to continue ongoing treatment during the study, with the assumption that psychotherapy and medication effects over an interval of 4 weeks were likely to be small and randomized between groups.

After signing written informed consent, volunteers, ages 60–79 years, with significant depressive complaints were evaluated for the randomized clinical trial. An intake assessment questionnaire included questions concerning depressive symptoms, the Geriatric Depression Scale (GDS) [41], the Horne-Östberg Morningness-Eveningness Scale [42], questions concerning sleep, questions concerning time in daylight, and questions concerning medication use and current illnesses. The *Structured Clinical Interview for DSM-IV Axis I Disorders (Non-patient Edition) *[43] was administered during the baseline week, prior to randomized treatment. For enrollment in the study, a GDS score of 11 (indicating probable major depression) [41] was required. This cut-off has 81% sensitivity and 61% specificity for DSMIII-R major depression. Meeting full DSM-IV criteria for current major depressive disorder was not required, because many aging depressed people are significantly troubled by minor depressive disorders without meeting criteria for major depressive disorder [44]. Any lifetime history of mania required exclusion of the potential volunteer, as a history of mania appears to predict a greatly increased risk of a manic switch during bright light treatment [45]. It may be assumed that almost all aging depressed volunteers will offer sleep complaints. The purpose of the sleep questions was to identify symptoms suggestive of circadian sleep phase disorders, such as evening drowsiness or prolonged sleep latency, early awakening or tending to awaken late, which might suggest particular responsiveness to bright light treatment. These symptoms were evaluated to select the optimal treatment, and their presence suggested that the volunteer was a good candidate for study recruitment. In contrast, symptoms strongly suggestive of sleep apnea such as obesity, loud snoring, and choking and gasping during sleep were contra-indications to selection, as light is not known to be useful for depressive symptoms related to breathing disorders in sleep. The questions concerning daylight exposure enabled us to avoid volunteers who were outdoors so much that light treatment had little to add (e.g. outdoors for more than an hour during times of potential light triggered circadian rhythm shifts, that is morning or evening hours). Throughout the five weeks of study, subjects continuously wore an Actillume wrist monitor (Ambulatory Monitoring Inc., Ardsley, NY) to record movement and light exposure.

Automated sleep scores were obtained from these records using a previously validated algorithm [46,47]. Available Actillume sleep measures for the night intervals in the baseline week (an average of 6.8 nights) and the final treatment week (an average of 6.7 nights) were averaged. Sleep parameters at baseline were contrasted among the groups assigned to bright and dim light in the morning, midday, or evening, in two-way factorial ANOVA. Further, sleep parameters at the end of treatment were likewise contrasted, using the baseline values as a covariate.

**Figure 1:**
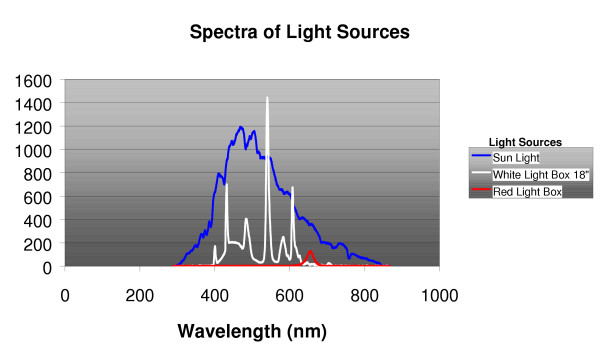
Spectrophotometric measures of illumination are shown comparing daylight with the white and red treatment lights. Sunlight was measured with the photometer pointed towards the horizon (and shaded from direct sun) near noon on a clear sunny day (32.85 North latitude, 2/2/05). White light was measured at 18" with the photometer oriented towards the center of the box. The red light was measured with the photometer adjacent to the diffuser, because at 18" the illumination was too dim to be plotted on the same scale. The irradiance scale was arbitrary (uncalibrated) but identical for the three measures.

Volunteers began placebo treatment during the initial baseline week of the study. The purposes of placebo treatment during the baseline week were to identify placebo-responders, to test the volunteer's compliance, and to collect baseline data. A member of the research staff visited the volunteer's home bringing a modified Sunbox light enclosure containing two red LED light sources (Enerlight Corp. Model ENS2000), producing less than 10 photopic lux of red light measured at 18" by a photometer pointed towards the center of the diffuser (See Figure 1). The volunteer was asked to sit in front of this dim-red-placebo light box for 60 minutes, at mid-wake, for the baseline week of the study. Mid-wake, determined from the questionnaires, was half-way between out-of-bed time and lights-out time.

If a volunteer's GDS score dropped 20% or more from the first day to the last day of the baseline week, the volunteer was dropped prior to randomization. This aspect of the design followed the general principle of clinical trial design that better contrasts between active and placebo treatments can often be obtained if early placebo-responders are eliminated. As a courtesy to those subjects who were dropped, they were permitted to continue with the dim-red-placebo light box for 4 weeks if they wished.

Volunteers underwent baseline circadian assessment during the week of dim-red-light placebo treatment. The investigators predicted the best-choice timing for each volunteer, in advance of treatment randomization, using subjective questionnaire, sleep log data and the Actillume recordings. Evening bright light was selected as the best choice for volunteers who reported that they were falling asleep in the evening excessively before going to bed or who found themselves going to bed earlier than desired, who complained of early awakening, or whose baseline light-activity recordings indicated that they had these problems. Conversely, early morning bright light was selected for volunteers who reported that they had trouble falling asleep at their desired bedtime, had a long sleep latency, had trouble waking up in the morning or whose baseline light-activity recordings suggested a delayed sleep phase. Mid-day bright light was selected for volunteers who reported no symptoms suggestive of a circadian sleep-phase disorder, who reported mixed symptoms which did not segregate into a consistent pattern suggestive either of advance or delay, and whose light-activity recordings gave no persuasive indication of a circadian-sleep-phase disorder. Where choice of assignment seemed inconclusive, the investigators sought to assign volunteers to equalize the numbers receiving morning, mid-day, and evening treatment. Utilization of placebo groups with each timing was necessary to control for the different sleep times of those assigned to the different treatment timings and for the behavioral influences of sitting in front of light boxes at different times of day.

Having been assigned one of three treatment times in advance, placebo non-responders within each assigned treatment time were then randomized into one of 2 treatment groups: A) 8,500 lux bright white light (model: SunRay, Sunbox, Gaithersburg, MD) or B) 10 lux dim-red-light placebo. Randomized assignment within blocks was stratified for time-of-treatment, age below or ≥ 68 years, and baseline GDS score below or ≥16 using computerized randomization and sealed envelopes.

To test the benefits of partial sleep deprivation, on the final night of baseline, we asked volunteers to awaken themselves 4 hours after going to bed and to remain awake for the second half of the night. They were asked to call our telephone answering machine every half hour to confirm that they were awake during that time period. In a previous study, we found such home sleep deprivations work well without complication [39]. An additional GDS rating was completed at the usual time of awakening after this half-night sleep deprivation.

As reviewed by Eastman [48], the issue of placebo responses has been a serious problem in clinical bright light studies, though the placebo problem has been negligible in studies of the physiologic effects of light. We have employed dim red light as a placebo, reasoning that because the light was dim and because the red part of the spectrum is relatively inactive biologically [49], there would be no substantial effect. Many subjects consider bright white fluorescent light to be glaringly ordinary, whereas the red light may appear more special. The investigators find no controlled evidence that dim-red-light is anything but placebo. Fortunately, claims by others of red-light benefits allowed us to tell volunteers, without deception, that some people think that red light is active (even though we do not agree). In this way, we attempted to maintain the best possible subject blind to the treatment expectation.

To assess subject perception of treatments, an expectation rating was obtained at the beginning of the study and at the end of treatment. This consisted of a 100 mm visual analog scale for the expectation for both sleep and mood improvement. The initial rating was obtained after the subject was randomized and had seen the light they would be using but before the first actual treatment. A final assessment was obtained on the last day of the study.

During the baseline (placebo) week, the volunteer completed sleep-activity logs daily and continuously wore an Actillume to monitor baseline sleep-wake patterns and baseline illumination patterns. The GDS [41] was obtained on the first and last days of the baseline week. Fractional urine samples supplemented by limited saliva collections were collected during baseline and final weeks to characterize the circadian phase of the subject's melatonin rhythms.

Urinary excretion of the major melatonin metabolite, 6-OH-melatonin sulfate (6-sulphatoxymelatonin or aMT6s) was used as the primary phase marker of the endogenous circadian pacemaker. For a 24-hour period prior to treatment and another at the end of four weeks of treatment, volunteers collected each fractional urine specimen, measured and recorded the time and volume, and froze duplicate 2 cc vials for assay. On the same occasions, from 4 hours before until bedtime and again from wake-up until 4 hours after wake-up, the volunteer collected and recorded the time of hourly saliva samples for a total of 5 evening samples, and 5 morning samples. Saliva samples were collected to provide a potentially more accurate measure of the onset and offset of melatonin secretion than could be interpolated from aMT6s excretion.

Urinary aMT6s was assayed using 96 well ELISA kits (Bühlmann Labs, EK-M6S) purchased from ALPCO, Ltd. (Windham, NH), a competitive immunoassay that uses a highly specific rabbit anti-6-sulfatoxymelatonin antibody and a second antibody capture technique. Assay performance has been extensively validated by the manufacturer and results correlate well with the Arendt (Stockgrand, Ltd) RIA (r = 0.987). Saliva samples were collected using polyester-swab Salivettes (Sarstedt, Numbrecht, Germany), centrifuged, and stored at -70°C. until assay. Samples were pretreated and assayed using Bühlmann laboratories Direct Saliva Melatonin ELISA kits (EK-DSM, ALPCO, ltd., Windham, NH).

Reliable estimates of aMT6s acrophase, onset and offset require a clear circadian pattern that is free of major irregularities. To ensure reliability, we examined all excretion curves visually to record an overall quality score for each 24-hour profile. This evaluation, blind to all other information on participants, was based mainly on the shape and completeness of the ng/h curve, but agreement between ng/h and ng/ml temporal patterns, regularity of the baseline, and reliability of the patient log were also considered. Based on this evaluation, the present analyses used 48 profiles from week 1 and 43 profiles from week 5 excluding 24% for poor quality (15 and 14 respectively). Aside from the above considerations, data were unavailable (assays were not performed) for 26% of cases due to the poor quality of the home collection or the loss of critical samples.

The aMT6s excretion rate for each urine sample was computed and transformed into 5 min epoch data and the resulting time series data were imported into Action3 software (Ambulatory Monitoring Inc., Ardsley, NY), where they were aligned with activity, sleep and illumination data and further checked for accuracy. Then 24-hour least-squares cosine fits were computed for each aMT6s collection, yielding mesors and acrophases. To estimate the duration of nocturnal aMT6s excretion, the onset and the offset of the excretion were estimated by interpolation of times at which the excretion rate (ng/h) crossed the mesor level. The time of onset of aMT6s excretion was estimated as the upward crossing and offset as the downward crossing of the mesor level. The aMT6s duration was defined as the interval between onset and offset times.

Salivary melatonin data were rated for reliability following guidelines similar to those used for urinary aMT6s, and poor quality data were eliminated from further analyses. Concurrently, where possible, onset was defined by the first data point in the evening that was elevated above a subjectively viewed baseline and was preceded by at least one point at or below that value. Similarly, offset was defined by the last data point in the morning that was elevated above the baseline and was followed by at least one point at or below baseline. Values used were an average from two blind raters. Due to problems with the quality of home collected saliva data, and some failed assays, good quality saliva melatonin data were available from only 28 collections in week 1 and 35 collections from week 5.

The primary test of the light treatment in correcting circadian phases was a contrast of the phase dispersion of aMT6s acrophases in the bright-light-treated group vs. the placebo group after 4 weeks of treatment, to test the hypothesis that light treatment reduces dispersion of circadian phases. This contrast was computed by calculating the median acrophase of each group. Then, for each subject in the trial, the [absolute value] deviation of each acrophase from the group median was computed pre- and post-treatment (d_1 _and d_2_), and the change in deviations was calculated (D = d_2 _- d_1_).

In addition to daily log sheets used to record activity, sleep behaviors, and visual analog self-ratings of mood, the subjects completed a weekly GDS and a Systematic Assessment for Treatment Emergent Events (SAFTEE) symptom scale [50]. Further mood measurements were made at baseline and end-of-treatment using the SIGH_SAD_SR, a self-rating form of the Hamilton Depression Rating Scale (HDRS) which includes atypical items previously shown to be responsive to light treatment [51]. Additionally, when a graduate student, blind to treatment, was available, an HDRS interview was administered at baseline and near the last day of the study.

Four weeks of treatment were carried out with weekly symptom assessments and continuous wrist recordings of activity and illumination exposure. The investigators visited subjects weekly to assure their safety and their compliance with the study, to administer and collect rating forms, and to transfer data from the Actillume recorders. A final symptom and circadian assessment was completed in the last 48 hours of the 4-week randomized treatment. Two-week, 4-week and 3-month follow-up assessments were obtained.

Records from the Actillume monitor indicating total activity, sleep-wake, log_10_[lux], and total lux were fitted to cosine curves for each subject. The mesors or fitted cosine means were examined, as well as the acrophases which indicate the time of day of the fitted peak.

### Healthy controls

Control subjects were recruited over the same interval as depressed subjects. Volunteers who did not have a history of depression were invited to participate as healthy controls. The informed consent and screening procedures were similar to those for depressed subjects. If qualified, the volunteers wore an Actillume monitor for 48 hours. During this period they collected urine and saliva samples over 24 hours in the same manner as the depressed subjects.

## Results

Based on power analysis, it was our intent to study 150 subjects over a five year period. After three and one-half years, an interim data analysis did not detect any trend towards a benefit from bright light (though a minimal trend appeared in the final results below). We convened an informal data safety and monitoring board to determine if continuing to collect data would be justified. Because demonstration of a significant light treatment benefit had become very unlikely, the board recommended discontinuing the study. Results after early termination are presented below.

Signed consents for 191 participants were obtained to conduct screening. Of these, 119 subjects met criteria for enrollment, of which 81 completed the protocol. About one third of the 119 subjects responded to placebo light and were dropped during the first week of the study, prior to randomization. There were 7 dropouts for other reasons, before randomization. Among these reasons were: family emergencies, discomfort with the research protocol and equipment, and a decision not to continue for lack of motivation. There were 2 dropouts after randomization: both were due to medical health issues, unrelated to the research, which required hospitalization. One had received bright light and the other received dim light. There were 34 male and 47 female subjects who completed the study. Bright light treatment was completed by 41 subjects, and 40 received dim red light. The mean age for the completers was 67.7 years (SD = 5.45) and ranged from 60 to 79.

Of the 81 subjects who completed the study, 24 were being seen by a psychotherapist during research treatment. Of these 24, 12 received bright light and 12 received dim light. In all 24 cases, treatment was stable during the study. Medication usage for the sample was as follows; Antidepressants = 30 (15 bright light, 15 dim light), Antianxiety = 11, Cardiac = 16, Antihypertensive = 25, Analgesic = 20, Hypnotic = 8, Thyroid = 15, Hormone Replacement Therapy = 22, Diabetes Drugs = 8 cholesterol-lowering drugs = 14. Nineteen subjects received both psychotherapy and psychiatric medication: 9 received bright light and 10 received dim light. Seven of the bright light subjects and 7 of the dim light subjects remained stable on their treatment regimen during the study. Two subjects receiving bright light had medication changes and 3 dim light subjects had medication changes, without any consistent patterns. Among all 81 subjects, those patients who were not taking antidepressant medication experienced nonsignificantly greater mood improvement from light treatment as measured by the GDS (p = .124), HDRS17 (p = .150) and, HDRS21 (p = .146). Thus less than half of our sample subjects were receiving psychotherapy and/or antidepressant medication. They represented a broad spectrum of depressed patients which ranged from long term chronic conditions with multiple episodes to single events which both did and did not meet criteria for Major Depressive Disorder. We obtained this sample deliberately as we wished to test the general applicability of light therapy for elders with depressive symptoms.

Based on the SCID interviews, Axis I diagnoses for the sample were as follows; Major Depressive Disorder 57, Minor Depressive Disorder 14, Adjustment Disorder 2, Schizoaffective Disorder 1, and Mood Disorder due to a General Medical Condition with depressive features 1. None of the subjects met criteria for Seasonal Affective Disorder. Five subjects had GDS ≥11 but did not meet SCID criteria for any Axis I diagnosis.

For the 81 subjects who completed the protocol, light treatments were randomized and stratified as shown in Table 1. Groups assigned to active and placebo light treatment were balanced in age and severity overall and by time-of-day of treatment. Expectations for sleep and mood effects of light treatment are shown in Table 2. Repeated-measures MANOVA showed no difference in the sleep and mood expectations between the bright and dim treatment groups either before or after treatment, suggesting that the dim red placebo induced balanced expectations.

**Table 1:** Randomization of Light Treatment Time and Stratification by Age and Depression Severity

**Time of Light Treatment**	**Bright**	**Dim**	**Total**
**Morning**	13	15	28
**Mid-Wake**	15	16	31
**Evening**	13	9	22
**Total**	41	40	81

**Age-Depression Severity Group**			

**Age<68, GDS<16**	4	4	8
**Age<68, GDS ≥16**	18	16	34
**Age ≥68, GDS<16**	6	6	12
**Age ≥68, GDS ≥16**	13	14	27
**Total**	41	40	81

**Table 2:** Expectations for Improvement in Sleep and Mood

100 mm Visual Analog Scale, 0 = Worse 100 = Better								
**Measure**	**Sleep**				**Mood**			

**Light**	**Bright**		**Dim**		**Bright**		**Dim**	

**Time**	**Initial**	**Final**	**Initial**	**Final**	**Initial**	**Final**	**Initial**	**Final**

**N**	40	40	38	38	40	40	38	38
**Mean**	71.4	71.2	68.7	63.5	72.2	73.4	72.3	68.3
**SD**	15.3	14.7	13.5	21.6	15.5	13.4	15	21

### Sleep changes

As might be anticipated from the phase-typing, subjects assigned to morning, midday, and evening light went to bed at 00:03, 23:12, and 22:49, respectively (p < 0.05). Similarly, they got out of bed at 08:14, 07:21, and 07:09 respectively (p < 0.06). Further, those treated with bright light got out of bed an average of 37 min. earlier (p < 0.06), regardless of treatment timing and without significant interaction with treatment time. There was no significant effect of randomized treatment assignment on time of going to bed or getting out of bed, nor did total time in bed vary significantly by treatment. The data for times of sleep onset and sleep offset within the nocturnal periods had similar trends. The total amount of Actillume-estimated sleep was balanced at baseline and not significantly affected by treatment assignment. The baseline, initial week, estimate of total sleep during the nocturnal period was 327 minutes. During the final week the estimate of total sleep during the nocturnal period was 330 minutes. The sleep efficiencies for the initial week and the final week were 70.8 and 72.6 respectively. Wake After Sleep Onset (WASO) did not vary by treatment time or randomization at baseline, but controlled for the baseline value, there was a marginal interaction of randomization with time of treatment at the end of treatment (P < 0.08), perhaps indicating that morning bright light tended to decrease WASO. However, the number of awakenings during the night and the sleep latency did not vary significantly either at the beginning or end of treatment by randomization or treatment timing.

### Mood improvement

Mean mood scores for the different groups at each measurement point are shown in Tables 3 and 4. There was little improvement in mood after 1/2 night of wake therapy (Table 3). Seventy-one subjects had some usable actigraphic data for week one and week two, but 10 were missing part of these data. Approximately 85% of the subjects phoned in every 30 minutes during the wake therapy period. Nine of these 71 subjects either did not attempt to wake early or were not able to remain awake for a significant portion of the planned wake therapy period. According to actigraphic scoring, twenty-four of 71 (34%) were able to remain 100% awake for the entire wake therapy period (4 hours); however, only 12 of these (17 % of the total) also remained 100% awake for the rest of the day. The mean percentage awake during wake therapy period for those attempting wake therapy was 87.5% (SD = 15.9, N = 62), whereas the mean percentage awake on a comparison night in the previous (baseline) week was 39.1% (SD = 20.8, N = 61). The mean percentage awake for the full wake therapy day (20 hours) was 88.2% (SD = 32.9, N = 61). There was no significant Spearman correlation of wake therapy compliance (percent awake during the wake therapy period or during the full wake therapy day) with the change in mood from baseline to the morning after wake therapy. Likewise, there was no significant correlation between wake therapy compliance and GDS mood score changes one week later, either for the entire sample or for bright or dim light-treated groups separately.

**Table 3:** GDS Scores by Week by Light Condition and Treatment Timing: Mean (SD) N

**Time Light**	**Morning**		**Mid-day**		**Evening**		**Total**	
	
	**Bright**	**Dim**	**Bright**	**Dim**	**Bright**	**Dim**	**Bright**	**Dim**
**Baseline start**	21.23 (6.08) 13	21.00 (4.47) 15	18.73 (2.91) 15	19.50 (4.43) 16	18.54 (5.63) 13	17.00 (4.27) 16	19.46 (5.01) 41	19.50 (4.56) 40
**End of baseline week**	21.77 (5.85) 13	20.33 (4.53) 15	18.93 (3.41) 15	19.63 (4.83) 16	18.62 (5.61) 13	17.89 (5.09) 9	19.73 (5.07) 41	19.50 (4.74) 40
**After wake therapy**	21.67 (5.68) 9	19.43 (6.73) 14	18.00 (5.31) 12	18.00 (5.85) 13	16.60 (5.97) 10	17.14 (4.14) 7	18.61 (5.82) 31	18.41 (5.85) 34
**Treatment Week 1**	20.00 (5.55) 13	19.33 (7.69) 15	18.20 (4.06) 15	16.94 (3.99) 16	15.08 (8.41) 13	15.11 (5.11) 9	17.78 (6.35) 41	17.43 (5.95) 40
**Treatment Week 2**	20.08 (6.22) 13	18.60 (7.60) 15	16.21 (5.69) 14	14.38 (5.30) 16	14.75 (8.07) 12	12.33 (5.45) 9	17.05 (6.87) 39	15.50 (6.65) 40
**Treatment Week 3**	18.85 (6.50) 13	18.50 (6.44) 14	15.00 (5.41) 15	14.81 (5.97) 16	13.23 (6.66) 13	13.00 (7.05) 9	15.66 (6.45) 41	15.72 (6.61) 39
**Treatment Week 4**	17.38 (6.96) 13	18.67 (7.79) 15	14.07 (6.06) 15	13.81 (5.60) 16	11.77 (7.00) 13	12.22 (6.53) 9	14.39 (6.88) 41	15.28 (7.07) 40
**Two-Week follow-up**	16.25 (7.96) 12	16.00 (7.72) 11	13.69 (5.41) 13	11.85 (5.80) 14	11.80 (6.66) 10	12.13 (8.41) 8	14.03 (6.77) 35	13.30 (7.18) 33
**Four-Week follow-up**	17.25 (6.54) 8	14.00 (9.03) 9	12.77 (5.69) 13	13.08 (5.27) 13	13.55 (6.25) 11	10.25 (8.40) 8	14.16 (6.18) 32	12.60 (7.29) 30
**3-Month follow-up**	14.00 (9.47) 8	18.14 (9.58) 7	15.91 (5.26) 11	11.21 (5.74) 14	16.29 (6.97) 7	11.60 (9.63) 5	15.42 (6.99) 26	13.15 (7.97) 26

**Table 4:** HDRS Scores by Week by Light Condition and Treatment Timing: Mean (SD) N

**Time Light**	**Morning**		**Mid-day**		**Evening**		**Total**	
	
	**Bright**	**Dim**	**Bright**	**Dim**	**Bright**	**Dim**	**Bright**	**Dim**
**Self Report HDRS 17-Baseline**	19.76 (8.46) 13	19.20 (6.05) 15	16.80 (6.12) 15	17.31 (5.53) 16	14.00 (6.22) 13	15.22 (3.38) 9	16.85 (7.18)41	17.55 (5.44) 40
**Self Report HDRS 17-Final**	15.46 (8.49) 13	13.27 (5.78) 15	10.87 (5.87) 15	10.38 (4.79) 16	6.92 (4.13) 13	9.55 (7.92) 9	11.07 (7.12) 41	11.27 (6.02) 40
**Blind HDRS 17-Baseline**	21.14 (7.86) 7	21.60 (6.35) 10	18.50 (5.40) 10	18.20 (7.30) 10	15.13 (8.18) 8	16.40 (3.78) 5	17.96 (6.95) 27	18.89 (6.35) 27
**Blind HDRS 17-Final**	16.00 (8.74) 7	11.80 (5.47) 10	10.70 (4.74) 10	12.30 (6.26) 10	6.62 (5.24) 8	9.20 (7.60) 5	11.15 (7.01) 26	11.38 (5.97) 26

Subjects' moods improved under both treatments at all times of day by treatment week 4. Combining the 3 times of day, GDS scores improved from 19.46 to 14.39 in those treated with bright light and from 19.50 to 15.28 in those treated with placebo dim light. However, there were no significant differences in treatment effects nor were there time-by-treatment interactions. There was no significant treatment effect for any treatment time. The average HDRS17 (extracted from the self-rated SIGH-SAD-SR) improved by 6 points. Combining the 3 times of day, HDRS17 scores improved from 16.85 to 11.07 in those treated with bright light and from 17.55 to 11.28 in those treated with dim light. Again there were no significant treatment effects or time-by-treatment interactions. Blind HDRS17 ratings, when available, were consistent with the self-rating (HDRS17) scores. A power analysis indicated approximately 81% power to detect an effect size of 0.32 in either the GDS or self-rated HDRS.

### Adverse reactions

Participants experienced no psychiatric hospitalizations, suicide attempts, or deaths during the study. However, one participant who dropped out while receiving bright light treatment died in the hospital due to late stage emphysema, 3 months after leaving the study. There were no incidents of mania or hypomania during the light treatment.

The weekly SAFTEE physical symptom inventory was examined for adverse reactions to both light treatments using Wilcoxon's Signed Rank Test. To improve the stability of measurement, the 94 individual symptoms were grouped into 17 SAFTEE-defined categories. The results of these group tests are contained in Table 5. The symptom groups for Head, and Other improved with bright light while Urination worsened, and with dim light, Mouth and Teeth improved while Urination, and Genital/Sexual Functioning worsened.

**Table 5:** SAFTEE Symptoms, Mean Scores for Beginning and End of Light Treatment with Wilcoxon Signed Ranks Test

**Light Condition**	**Bright**			**Dim**		
**Symptom Category**	**Baseline**	**Post Treatment**	**p**	**Baseline**	**Post Treatment**	**p**

**Head**	5.62	4.92	**.012**	5.35	5.14	.065
**Eyes**	8.53	8.61	.404	8.76	8.27	.168
**Ears**	5.15	5.22	.791	5.14	5.53	.502
**Mouth/Teeth**	8.48	7.82	.193	8.41	7.28	**.001**
**Nose/Throat**	6.71	7.03	.297	6.58	6.12	.312
**Chest**	8.17	7.74	.683	7.71	8.00	.315
**Heart**	2.28	2.19	.276	2.69	2.55	.106
**Stomach/Abdomen**	6.03	5.46	.080	5.37	5.00	.239
**Bowel**	8.64	8.40	.487	8.39	8.32	.790
**Appetite**	7.91	7.58	.394	7.69	7.66	.699
**Urination**	5.79	7.11	**<.001**	6.28	8.18	**<.001**
**Gynecology**	9.40	9.00	.854	8.91	9.75	.655
**Genital/Sexual**	8.31	9.64	.077	7.85	9.87	**.004**
**Muscle/Bone**	5.97	5.85	.573	5.25	4.94	.178
**Walking/Moving**	7.06	7.36	.271	7.21	7.74	.239
**Scalp/Skin**	5.76	5.70	.636	5.39	5.53	.120
**Other**	28.73	23.4	**<.001**	27.32	25.17	.091

Testing with the Mann-Whitney U-test showed there were no significant differences in change scores (baseline minus post treatment) of the 94 individual SAFTEE items between bright and dim treatment. Of the 17 symptom groups, only one showed a significant treatment difference, a greater improvement in "Other" in the bright light group than in the dim light controls, (N = 47, p = 0.04, uncorrected for multiple testing.) The "Other" category contains a large number of mood related items (see Table 6).

**Table 6:** Mean SAFTEE "Other" Scores Before and After Light Treatment

**Other, Baseline average – Bright**	28.73
**Other, Baseline average – Dim**	27.32
**Other, End-of-treatment average – Bright**	23.39
**Other, End-of-treatment average – Dim**	25.17

Mann-Whitney (N = 47, p = 0.04, uncorrected for multiple testing)

### Measures of urinary aMT6s and saliva melatonin

The aMT6s mesors were not significantly different between patients and controls nor between genders, but a marginal interaction was observed in log_10 _[mesor] between gender and patient vs. control (DF = 1,88, F = 4.3, p = 0.043), with male patients having over 4 times the aMT6s mesor, whereas female controls had slightly higher mesors. Baseline salivary melatonin onsets and offsets and aMT6s acrophases, onsets and offsets tended to be later among patients than controls, but none of these differences were significant after adjustment for age and gender, nor were durations of secretion or excretion different. At baseline, aMT6s acrophases did not differ by treatment or time of treatment or their interaction, nor did acrophase shifts from baseline to the end of treatment. However, in females aMT6s acrophase was more phase advanced during randomized treatment (p < 0.05). Salivary melatonin offsets (not onsets) showed significant treatment, time of treatment, and interaction effects (greatest advance in morning-treated subjects receiving bright light), but the high-quality usable cases in each cell were as few as 2 (See Table 7). The Horne-Östberg Morningness-Eveningness scores for patients and healthy controls are reported in Table 7.

**Table 7:** Timing of Saliva Melatonin and Urinary aMT6s at Baseline Mean (SD) N

Times are in decimal hours			
**Saliva Melatonin**	**Depressed**	**Healthy Controls**	**Sig. (T-test, p)**

Onset	21.48 (1.67) 28	20.36 (1.98) 22	0.16
Offset	8.76 (2.14) 32	8.78 (1.22) 16	0.873* unequal variance
Duration	11.19 (1.64) 25	11.75 (1.75) 15	0.574
**Urinary aMT6s**			
Onset	23.95 (3.15) 48	22.22 (2.44) 28	**0.015**
Offset	9.72 (2.82) 48	8.70 (2.02) 28	0.099
Duration	9.75 (2.48) 48	10.48 (2.21) 28	0.201
Acrophase	4.25 (2.56) 49	3.36 (1.75) 28	0.107
Mesor (ng/hour)	652.12 (911.31) 49	485.40 (377.80) 28	0.36
**Horne-Östberg**	54.75 (11.45) 81	67.65 (9.38) 23	**< 0.001**

### Correcting circadian phases

Testing the hypothesis that light treatment reduces dispersion of circadian phases did not show any statistically significant difference in the pre- and post-treatment deviations (d_1 _and d_2_) from group median acrophases for aMT6s, sleep or activity. Light acrophase dispersion showed a significant effect of treatment time (p = .017). The difference in light acrophases was independent of the light condition (dim vs. bright). Therefore, it was more consistent with spontaneous regression than any treatment effect.

### Sleep-wake acrophases

For the five-week study period, regression slopes were calculated for the acrophase changes in light, activity and sleep. These slopes were compared (ANOVA) to differences between the week 1 and week 5 acrophases. The regression slope of the 5 weeks was similar to the week 1 minus week 5 results (data not shown).

There was a significant (p = 0.001) advance in illumination acrophase across the three treatment times, from baseline to the end of treatment, for the bright light group and not for the dim light group. Overall, the bright and dim light conditions did not differ significantly in light acrophase. The illumination acrophase change, week 1 minus week 5, showed a significant interaction between treatment time and treatment condition (p = 0.001) in the expected directions: that is, the group treated with bright light in the morning advanced acrophases more than the placebo morning group, whereas this did not occur with evening treatment. This demonstrates that light acrophase shifted in the expected direction in response to the morning bright light treatment. This change in light acrophase was associated with significant differences in the activity (p = 0.003) and sleep (p = 0.001) acrophase shifts between the bright and dim treatment groups but no significant treatment versus time interaction.

Although an objective measure of compliance was not determined, observation of the light records from the Actillume data suggested a high degree of compliance with bright light treatment. Dim light treatment could not be detected from the Actillume data. The self-reports of treatment time and duration were almost entirely consistent with instructions. The low dropout rates and the compliance of participants with wearing Actillumes and completing logs and questionnaires also suggested that compliance with treatment was probably high.

### Healthy controls

The healthy control group was active earlier, slept earlier, and reported significantly greater morningness but received less light than the depressed group at baseline (see Table 9).

**Table 9:** Baseline Activity, Light, and Sleep Comparing Depressed and Control Groups

**Measure**		**ACTIVITY**		**LIGHT**		**SLEEP**	
**Baseline**		**MESOR**	**ACROPHASE DEGREES**	**MESOR**	**ACROPHASE DEGREES**	**MESOR**	**ACROPHASE DEGREES**

**Depressed**	**Mean**	12.85	217.63	0.99	211.83	0.27	54.53
	**N**	78	78	78	78	78	78
	**S.D.**	4.46	26.05	0.26	19.47	0.08	26.05
**Healthy**	**Mean**	14.54	200.44	0.76	206	0.26	37.54
	**N**	28	28	28	28	28	28
	**S.D.**	3.94	15.66	0.28	9.52	0.05	13.65

**ANOVA**	**Sig. (p)**	0.079	**0.001**	**< 0.001**	0.132	0.704	**0.001**

## Discussion

Apart from an advantage on one scale of the SAFTEE side effects inventory (which would not be significant by Bonferroni criteria), bright light treatment had no advantage over dim placebo light. The beneficial effects found in the study might be attributed to several factors that were common to the treatment and control groups. The "placebo" effect, chiefly positive expectations, positive staff contacts, and spontaneous remission may have contributed to positive responses. Weekly visits, even with minimal social interaction, could have a positive effect. In addition, the social structure and regularized sleep provided by the protocol might be beneficial. An hour a day engaging in a treatment, thought to be helpful, may have induced a reduction in depressive symptoms. Participants with ongoing pharmacologic or psychotherapeutic treatment (possibly associated with greater severity or chronicity of illness) improved somewhat less than other participants, so such treatment was probably not a substantial confound.

As a result of the phase-typing treatment assignment, a group going to bed and arising later were assigned to morning light, and a group going to bed earlier were assigned to evening light, with the midday group intermediate. There was little evidence for the anticipated effects of the bright light treatment, which had been expected to alter sleep timing. Likewise, the effects of bright light treatment on melatonin phase were modest. Therefore, we would suspect either that these depressed elders were resistant to bright light effects or that we were less successful in obtaining compliance than we realized.

Even though this study is among the longest treatment trials investigating the effects of light on depression, it may be that the duration of treatment was not sufficient for this age group. A longer period may be needed for an elderly population with chronic depressive symptoms. Although the use of home based treatment may have given rise to less treatment compliance, these subjects did not require hospitalization clinically. Inpatient studies of light treatment have demonstrated antidepressant effects. To be cost effective for patients with milder depressions, light treatment must be feasible in the home. If compliance was a problem in our trial, it would probably be a similar problem in clinical application of this treatment for this age group.

The antidepressant response to placebo light treatment in this study was similar to that reported for placebo in drug studies [52]. However, placebo response is significant and needs to be considered as a confound in antidepressant light treatment studies. Removing placebo responders is common in pharmacology trials but has not commonly been practiced previously in light studies. Removing placebo responders from sample groups may decrease the placebo effects in the randomization portion of clinical trials, but even so the subsequent placebo effects were quite large. It is not evident that removal of placebo-responders was advantageous in this design. Exclusion of bipolar participants effectively prevented hypomanic adverse events, but possibly bipolar depressives are more light responsive.

Treatments were balanced by age and severity of depression, validating that the stratification randomization procedures were successful. The design was successful in balancing expectations between bright light and placebo. The similarity of blind-observer HDRS ratings with self-rating results tends to reduce concern with the blinding issue.

Only a small minority of elderly participants in this study were able to wake themselves up in the middle of the night and remain entirely awake until the next bedtime at home. Because study participants had a very low dropout rate and complied very well with other aspects of the protocol, it would appear that the poor compliance was due to the difficulty of the assignment rather than to poor motivation. The small number of subjects who were able to successfully remain awake for the entire prescribed time may explain why there was no detected antidepressant benefit of the wake therapy. The literature suggests that even a short nap may reduce the mood improvement obtained from wake therapy [53]. Unfortunately, in this age group, the tendency to fall asleep during the day was surprisingly strong [54,55]. Home wake therapy may be too difficult for elderly depressed patients. In addition, there is not much of evidence that sleep deprivation is useful in this age group [56,57]. Indeed, a recent study suggested that in this age group, sleep deprivation may actually interfere with antidepressant treatment [58], which is a possible explanation for the lack of success of this trial.

Tables 7 and 9 indicate that the rhythms of melatonin, sleep, and activity all peaked later (were delayed) as compared to the normal control group. Although this finding is not consistent with the older theory that aging depressives are phase-advanced, it is generally consistent with more modern results in post-menopausal women [32,59]. Table 9 indicates that mesor illumination (the daily amount of illumination) was significantly higher in the depressed participants than controls, which indicates that the relative baseline phase delay of depressives cannot be ascribed to below-average light exposure. However, since increased light exposure is generally associated with more advanced rhythms (as was observed comparing the bright light and placebo groups in this study), the results are consistent with the possibility that these depressed patients were subsensitive to circadian effects of light. The data in Table 8 show that the morning and evening bright light treatments tended to have the predicted phase-shifting effects, but only to a quite modest extent not significant in the interaction effects for activity and sleep. The lack of greater phase-shifting effects related to the experimental treatments provides an additional indication that either these depressives may have been subsensitive to bright light or their compliance may have been less than we realized.

**Table 8:** Acrophase Changes (Minutes) by Light Condition and Treatment Timing: Mean (SD) N

**Acrophase Difference (minutes) Baseline minus Final**	**Morning**		**Evening**	
	
	**Bright**	**Dim**	**Bright**	**Dim**
**Illumination**	80.69 (69.14) 9	1.53 (39.72) 14	-41.99 (63.48) 11	-28.77 (56.96) 7
**Activity**	25.84 (40.35) 8	-32.42 (60.00) 13	-0.96 (34.46) 11	-40.88 (48.03) 7
**Sleep**	39.59 (34.81) 9	-25.90 (42.96) 12	-3.80 (34.74) 11	-24.86 (48.32) 7

**ANOVA Sig. (p)**		**Illumination**	**Activity**	**Sleep**

**Treatment Condition**		0.485	**0.003**	**0.001**
**Treatment Time**		**0.001**	0.485	0.243
**Condition by Time**		**0.001**	0.128	0.054

Note: Positive change is earlier, negative change is later, at the end of treatment.

There was considerable overlap in baseline activity and aMT6s phases among the groups assigned to morning, mid-day, and evening bright light. Thus, our prospective phase-typing was imperfect. Nevertheless, examining the subgroups who were phase-typed correctly did not indicate any better responses to the bright light treatment.

## Conclusion

Antidepressant response to bright light treatment in this age group was not statistically superior to placebo. No timing of bright light treatment was significantly better than placebo or consistently better in self-ratings and blind ratings. There was minimal evidence for a relative reduction of diverse SAFTEE complaints in the bright-light-treated group. Contrary to what has been suggested by previous light studies [15,27], the trend was for evening light to appear at least as successful as morning or mid-day light. Were it a statistically significant difference, an advantage of evening light would be unexpected in a group of depressed participants who are generally more phase delayed than healthy individuals. We do not know whether the lack of light response found in this study might be associated with the age of the subject group, the relative mildness and chronicity of their symptoms, inadequate duration of bright light treatment, perhaps undetected compliance problems occurring in home treatment, or some aspect of the trial design such as the sleep deprivations. Considering the general evidence that light treatment is useful for depression, including in this age group, further testing with altered designs might be fruitful.

Upon early termination of this study, a trial of green light was initiated with somewhat more promising results. These results are presented in a separate report 60.

## Competing interests

The author(s) declare that they have no competing interests.

## Authors' contributions

RTL coordinated and carried out the clinical trial, performed statistical analyses, and drafted the manuscript. DFK conceived and drafted the design, administered and participated in data collection, and participated in statistical analyses and manuscript preparation. JAE carried out the immunoassays and contributed to statistical analyses and manuscript preparation. NCK carried out subject recruitment and collection and scoring of Actillume recordings and subject questionnaires. MAG constructed a software database and administered blind HDRS ratings. All authors reviewed and approved the final manuscript.

## Pre-publication history

The pre-publication history for this paper can be accessed here:


